# Role of necroptosis and immune infiltration in essential thrombocytosis

**DOI:** 10.1186/s41065-025-00428-1

**Published:** 2025-04-14

**Authors:** Guangming Li, Ying Guo, Yuanyuan Zhang

**Affiliations:** 1 Surgery Base Training, Shanghai Fengxian District Central Hospital, Shanghai, 201499 China; 2 Department of Hematology, Daqing Oilfield General Hospital, Daqing City, Heilongjiang Province 163001 China; 3 Internal Medicine Base Training, Shanghai Fengxian District Central Hospital, No.6600, Nanfeng Highway, Nanqiao Town, Fengxian District, Shanghai, 201499 China

**Keywords:** Essential thrombocythemia, Necroptosis, Immune infiltration, Multiple machine learning methods, Differentially expressed genes

## Abstract

**Background:**

Necroptosis, a recently identified form of programmed cell death involved in the pathogenesis of a variety of tumor and non-tumor diseases. Nevertheless, the function of necroptosis in essential thrombocytosis (ET) remains unclear, which is a classic myeloproliferative tumor.

**Materials and methods:**

The role of necroptosis in ET was determined via bioinformatics combined with qRT-PCR analysis of clinical samples. GSE57793 and GSE26049 datasets were recruited to identify necroptosis differentially expressed genes based on differential gene identification, necroptosis gene sets and data machine learning. Enrichment analysis (GSEA) was used to evaluate the gene enrichment signaling pathway of ET, immune infiltration analysis was used to explore the abundance of immune cell infiltration in ET, and the correlation between necroptosis differential genes and immune cell infiltration was studied.

**Results:**

Five necroptosis genes were recognized to be remarkably enriched in the necroptosis pathway, including CHMP1B, FTH1, HSP90AB1, IL1A, and RBCK1. The imbalance of invasion of Th1/Th17 cells was identified in ET, and the differential necroptosis gene was positively correlated with the infiltration of multiple immune cells. There is significant necroptosis in ET, which is enriched in the necrotizing apoptotic pathway, and is associated with immune infiltration.

**Conclusions:**

Necroptosis might drive the progression of ET via stimulating immune infiltration and immune responses. The findings bring new insights into the treatment mechanism and treatment strategy of ET in the future.

**Supplementary Information:**

The online version contains supplementary material available at 10.1186/s41065-025-00428-1.

## Background

Essential Thrombocythemia (ET), a chronic myeloproliferative disease, is characterized by an increased incidence of thrombocytosis, thrombosis and cardiovascular events [1]. The symptoms of ET are heterogeneous, and the lurking threat of secondary myelofibrosis and acute leukemia cannot be ignored. In the past few years, with the discovery of mutated genes in JAK2 (in 60%), MPL (in 3%) and CALR (in 20%), early diagnosis of ET has gradually become possible [2]. Existing pharmacotherapeutic approaches for ET are incapable in attaining a cure or prolonging the survival duration. Fortunately, the prognosis for ET is comparatively favorable, as the estimated median survival periods for younger patients are 33 years and 24 years, respectively [3]. However, a certain number of patients might experience severe symptoms, leading to a decline in their quality of life, which is manifested by a continuous deterioration. This is precisely what accounts for the diminished survival rate [4]. In view of this situation, ET patients require more effective and accurate diagnostic methods and even therapeutic targets.

In the past decade, immune infiltration has been emerged as a pivotal area of research, yielding a series of remarkable breakthroughs in the research of diverse diseases [5]. In the context of tumors, for instance, infiltrating macrophages in tumors are divided into two different subpopulations, activated by different polarized cytokines. M1 macrophages principally exert antitumor functions through the mediation of antibody-dependent cytotoxicity and the production of ROS and tumor necrosis factor. In contrast, M2 macrophages exhibit tumor-promoting activity by promoting tumor angiogenesis, immunosuppression, cancer cell invasion and metastasis [6]. Alzheimer’s disease is the most common form of progressive dementia in which infiltration of peripheral natural killer cells (NK) is also observed [7]. However, it is unknown whether the occurrence and development of ET also involve the infiltration of immune cells.

Necroptosis represents a form of programmed inflammatory cell death. Initially, it was identified as alternative mode to apoptosis upon binding to death domain receptors. Functionally, it can be regarded as a safeguard mechanism of the body against some pathogen invasions [8, 9]. It is characterized by a typical death receptor composed of a threonine protein kinase 1,3 (RIPK1, RIPK3)-MLKL mediated necroptosis pathway [8], which combines some features of necrosis and apoptosis, including membrane integrity disruption, organelle swelling, cytolysis, intracellular component leakage, etc [10]. The relationship between necroptosis and tumorigenesis and development is intricate, as it exerts a dual role in tumor microenvironment. At present, there is evidence indicating that necroptosis can promote the invasion and metastasis of breast tumors in mice, and eliminating MLKL to block necroptosis can significantly reduce lung metastasis in breast cancer lines [11]. However, the downregulation of another key member, RIPK3, is associated with low survival in acute myeloid leukemia, suggesting its antitumor effects through facilitating RIPK3-MLKL-mediated necroptosis and the differentiation of leukemia-initiating cells [12]. Necroptosis has also been implicated in other non-neoplastic diseases. For instance, following ischemic stroke, silencing RIPK1 or RIPK3 can polarize microglia and macrophages to the M2 phenotype, thereby exerting anti-inflammatory effects. In pancreatitis, the death of acinar cells can be prevented by silencing RIPK1, thereby reducing the severity of pancreatitis [13]. However, the role of necroptosis in ET has not been studied.

In this research, a comprehensive bioinformatics analysis was systematically conducted by utilizing the Gene Expression Omnibus (GEO) database. The aim was to provide an overview of immune infiltration in ET and explain if and how necroptosis contributes to ET development. In addition, the underlying biological mechanism of ET necroptosis was explored, and the association between necroptosis and immune cell infiltration was analyzed, so as to better understand the potential immune infiltration process during ET development. Finally, the expression of necroptosis genes in ET was verified by qRT-PCR.

## Methods

### Date retrieval and processing for this study

The Gene Expression Omnibus (GEO) data numbers GSE57793 and GSE26049 datasets were selected as data sources for this study. GSE57793 contains 16 Essential Thrombocythemia (ETs), which are divided into 8 ETs before IFNalpha2 treatment and 8 ETs after treatment. And 8 pre-treatment ETs were selected for research. GSE26049 contains 19 ETs and 21 control subjects. The 2 databases include a total of 27 ETs and 21 control subjects. 159 necroptosis were collected in the profiles of 159 genes related to necroptosis from the Kyoto Encyclopedia of Genes and Genomes (KEGG) Pathway databases (https://www.genome.jp/dbget-bin/www_bget?pathway+hsa04217). R version 4.2.2.

### Database consolidation and ET variance analysis

The data were removed from the batch effect using the R packages “limma” and “sva”, and the data were combined for a total of 48 samples and 21,640 genes. Differential genes were screened using adjust *P* < 0.05 and R package “limma” was used for differential analysis. Use the R packages “FactoMineR” and “factoextra” to plot.

### Gene set enrichment analysis (GSEA) of differential genes

Genes were GSEA using the R package “clusterpofiler”, including the Gene Ontology (GO), Kyoto Encyclopedia of Genes and Genomes (KEGG), and the Reactome database.

### Gene interaction analysis

The difference analysis was performed on the intersection of up-regulated and down-regulated genes and necroptosis genes, respectively. Protein interactions between intersecting genes were analyzed using the STRING database (https://cn.string-db.org/) [14]and the genemania database (http://genemania.org/) [15]. Machine learning using data was used to identify marker genes, including lasso regression using R-package “glmnet” and random forest machine learning using R-package “randomForest”. LASSO is a regularization method that can effectively perform feature selection by shrinking some coefficients to zero, which helps in reducing the dimensionality of the data and identifying the most relevant features. Random Forest is an ensemble learning method that builds multiple decision trees on different subsets of the data, which is robust to overfitting and can provide information about the importance of each feature. The R package “circlize” is used to perform genetic correlation analysis on necrotic apoptosis genes selected by machine learning. The R package “pROC” is used to construct ROC curves that genetically predict the onset of disease.

### Immune infiltration analysis

Based on machine learning, 5 core necroptosis genes were identified, and the infiltration of multiple immune cells was evaluated using the ssGSEA function of R-pack “GSVA”. Pearson correlation coefficient was used to evaluate the correlation between marker genes and various immune cells (only immune cells with *P* < 0.05 are shown, and R-pack ggplot2 is plotted).

### Exploration of core gene regulatory mechanisms

Prediction of 5 genes upstream miRNA and TF based on the Regnetwork database (https://regnetworkweb.org/) [16] was visualized using Cytoscape software.

### Patients and tissue samples

A total of 20 blood samples (including 10 ET and 10 control samples) were collected. Blood samples are stored at 4 °C for subsequent validation experiments. This study received approval from the Ethics Committee, and written informed consent was acquired from every subject.

### Quantitative reverse Transcription-PCR (qRT-PCR)

Whole blood samples were treated with Trizol. Total RNA was reverse transcribed by means of the Evo M-MLV RT Mix Kit with gDNA Clean for qPCR (AG11728, Accurate Biotechnology, Hunan, China, Co., Ltd). Subsequently, the SYBR Green Premix Pro Taq HS qPCR Kit (AG11718, Accurate Biotechnology, Hunan, China, Co., Ltd) was employed for qRT-PCR. The relative expression of the gene was analyzed by applying the 2^−ΔΔCt^ method and was normalized against GAPDH. Primer sequences are shown in Table S1.

### Statistical analysis

Data analysis was carried out using SPSS 21.0 software, while GraphPad 7.0 software was employed for figure generation. The data were presented as mean and standard deviation (SD). Student’s t test was utilized to compare the differences between the two groups. A difference was considered statistically significant when the *P* value was below 0.05.

## Results

### Identify differential genes

**Fig. 1:**
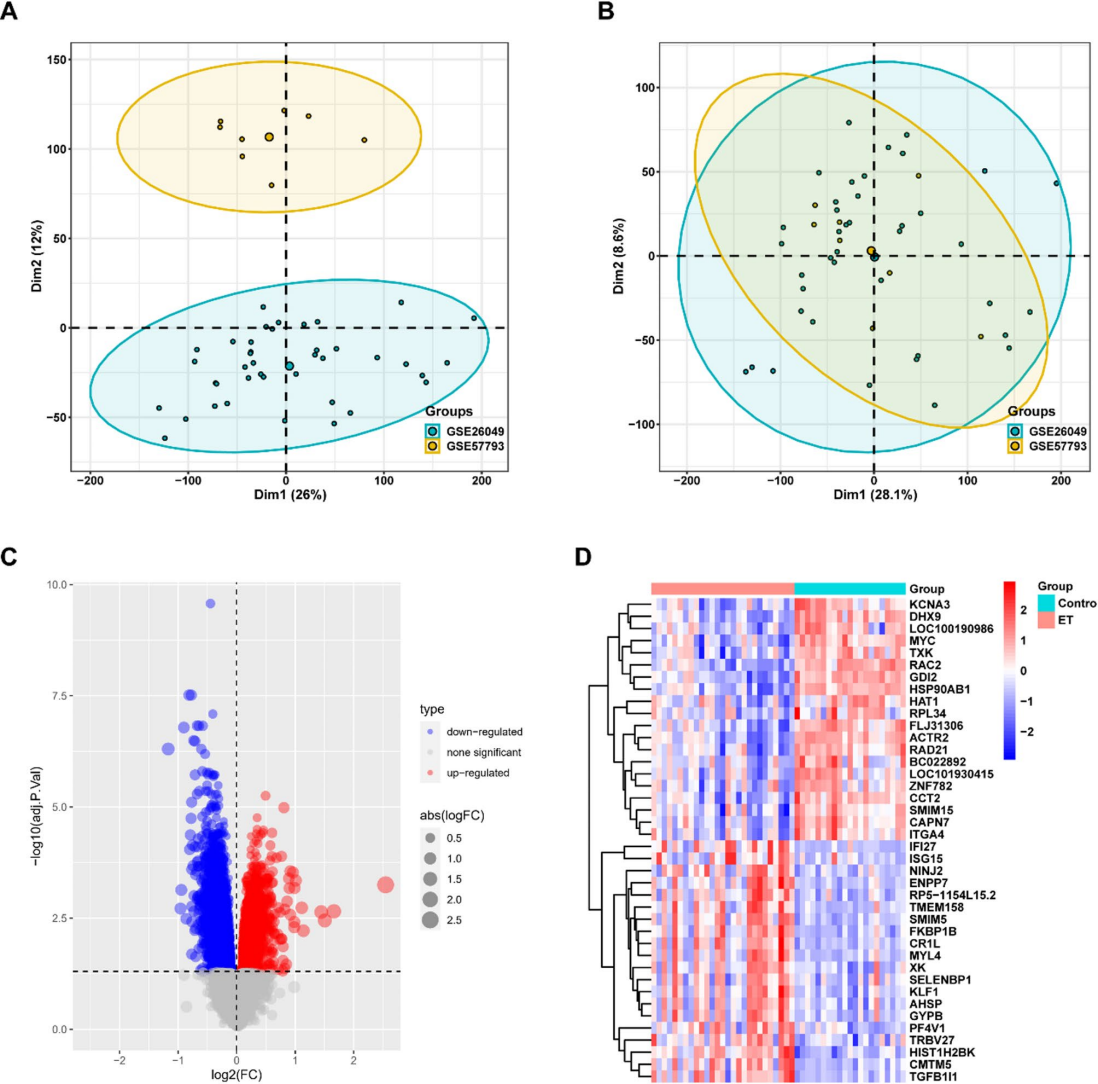
Data processing and analysis of differences between groups. PCA plot before data consolidation (**A**) and PCA plot (**B**) after data merge. Differential analysis volcano map (**C**) and differential analysis heat map (**D**)

Due to the differences in data sequencing batches and sequencing platforms between the two datasets, we first combined the data with a de-batch effect. The comparison results of data before (Fig. 1A) and after the combination (Fig. 1B) indicated that all experimental samples could be fully included. After the two datasets were combined, the 27 ETs and 21 control subjects included were analyzed for genetic differences. The volcano map (Fig. 1C) shows all the differential genes that meet the screening conditions, and the heat map (Fig. 1D) summarizes the distribution of the top 20 genes for differences between groups for easy visualization.

### Differential gene enrichment analysis

**Fig. 2:**
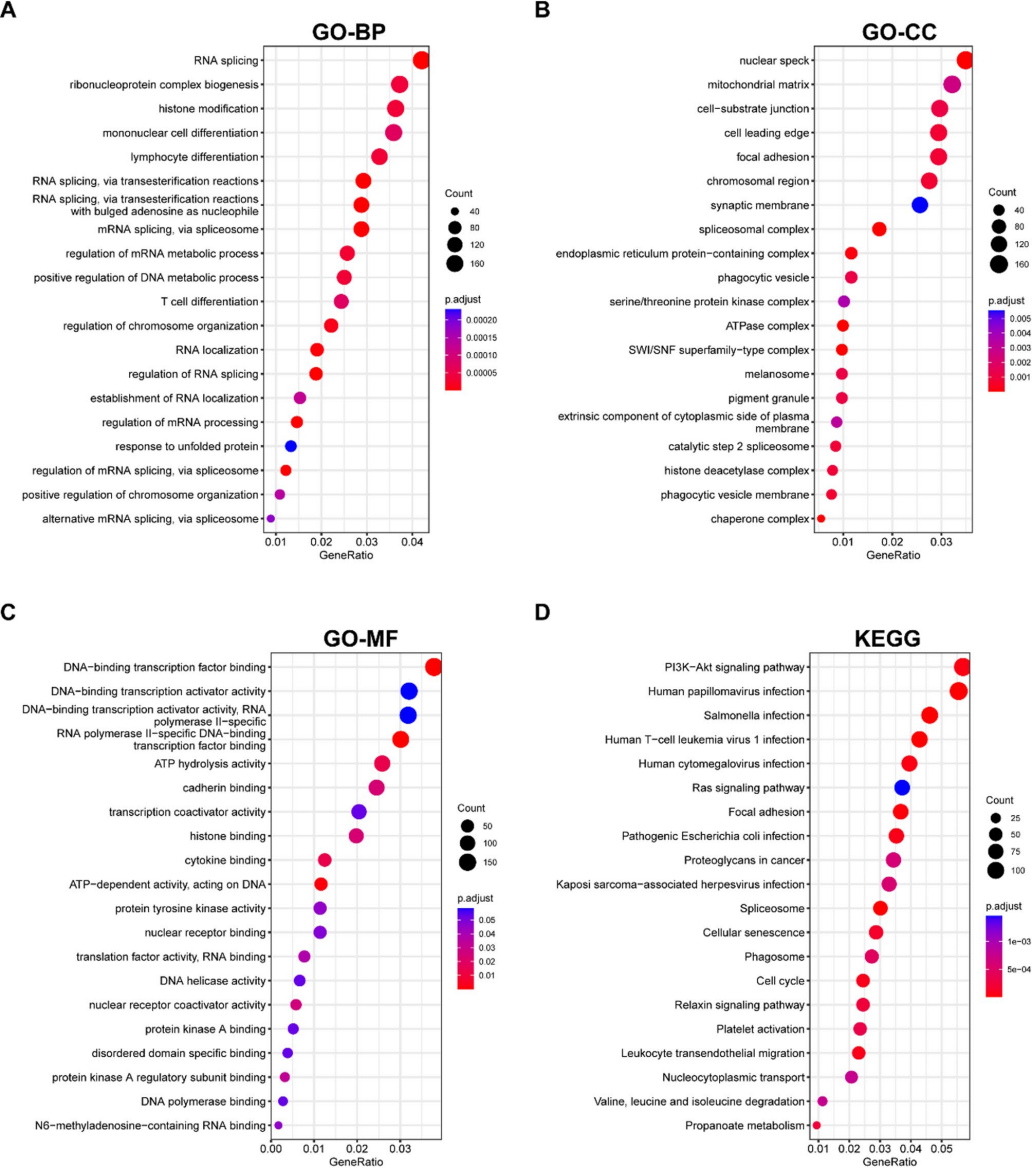
Differential gene enrichment analysis. GO database enrichment analyses include BP(**A**), CC(**B**) and MF(**C**). KEGG database enrichment analysis display(**D**). The correlation strength is positively correlated with the size of the point, and the color of the point represents the P-value, and the redder the hue, the higher the P-value. GO: Gene Ontology; BP: Biological Process; CC: Cellular Component; MF: Molecular Function; KEGG: Kyoto Encyclopedia of Genes and Genomes

Enrichment analysis of differentially expressed up- and down-regulated genes was performed using GO, and KEGG database to identify major signaling pathways. GO feature enrichment analysis includes Biological Process (BP) (Figs. 2A), Cellular Component (CC) (Figs. 2B) and Molecular Function (MF) (Figs. 2C). KEGG (Fig. 2D) enrichment analysis showed that the differential genes were mainly enriched in P13K-Akt, human tumor virus infection, salmonella infection, human T-cell leukemia virus 1 infection and human cytomegalovirus infection.

### Links of necrotizing apoptotic genes to ET

**Fig. 3:**
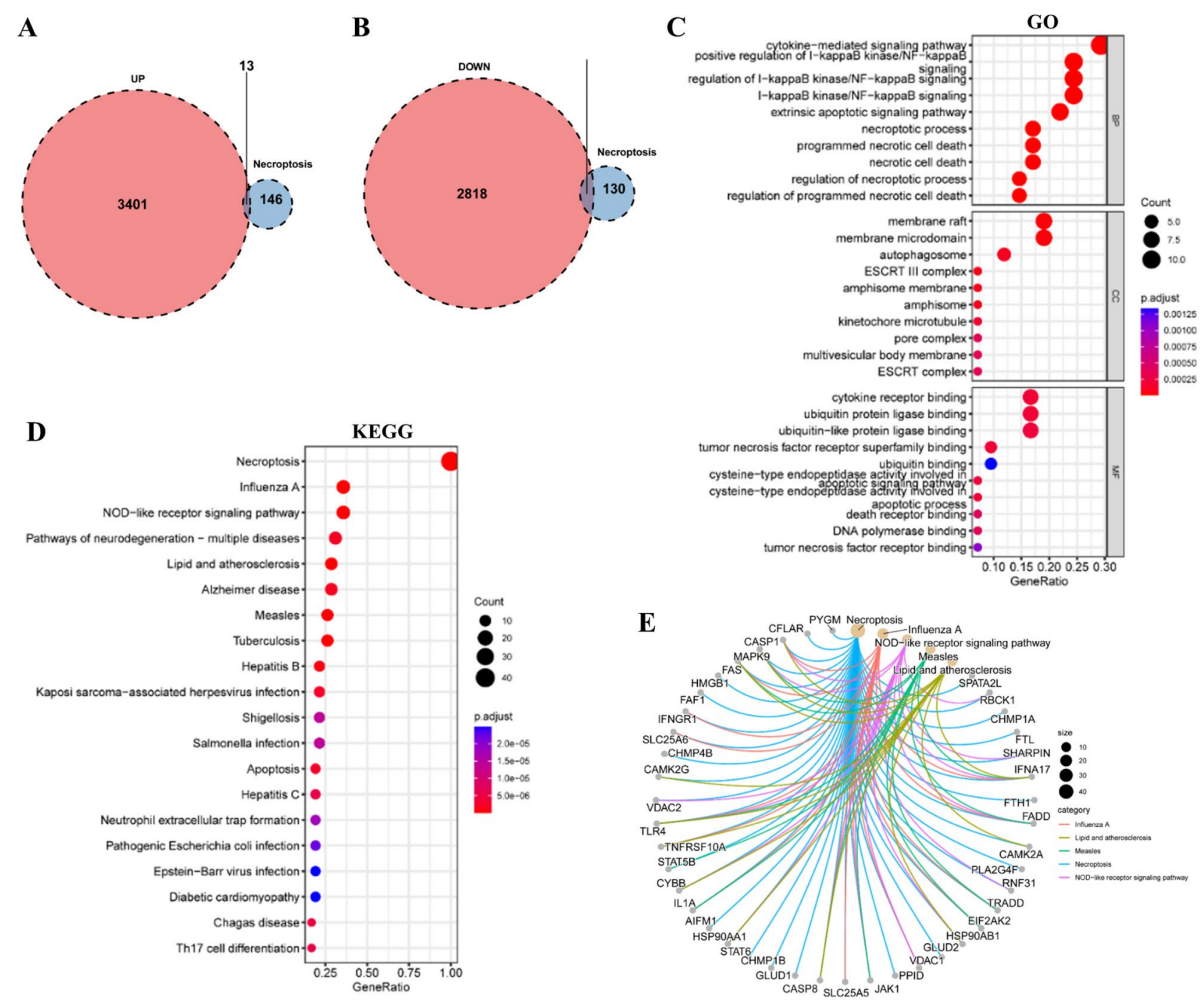
Genetic study of necrosis associated with ET. Differential analysis intersected upregulated genes with necroptosis genes (**A**) and differential analysis underregulated genes intersected with necroptosis genes (**B**). The 42 necroptosis genes expressed differently were analyzed by GO and KEGG enrichment (**C**, **D**), and the correlation intensity was positively correlated with the size of the dot, and the color of the dot indicated the P value, the redder the hue, the higher the P value. (**E**) KEGG results showed that the top5 pathway corresponded to 42 necroptosis genes. ET: Essential Thrombocythemia; GO: Gene Ontology; BP: Biological Process; CC: Cellular Component; MF: Molecular Function; KEGG: Kyoto Encyclopedia of Genes and Genomes

According to the outcomes of the differential analysis, we intend to further examine the role of necrotizing apoptotic genes in ET. The gene intersection analysis of 159 necroptosis genes and differential analysis of up-regulated and down-regulated genes showed that the expression of 13 apoptotic genes was upregulated (Fig. 3A) and the expression of 29 apoptotic genes (Fig. 3B) was down-regulated. Further GO (Fig. 3C) and KEGG enrichment analysis of 42 genes. KEGG (Fig. 3D) found that most of the genes were mainly enriched in necroptosis, influenza A, NOD-like receptor signaling pathway, neurodegenerative pathway - multiple diseases and lipid and atherosclerosis. The network diagram summarizes the correspondence between top 5 signaling pathways and 42 necroptosis genes (Fig. 3E). The above studies show that the onset and progress of ET may be related to necroptosis, which may be closely related to the invasion of multiple pathogens.

### Construction of differential necrotizing apoptotic gene protein interaction network

**Fig. 4:**
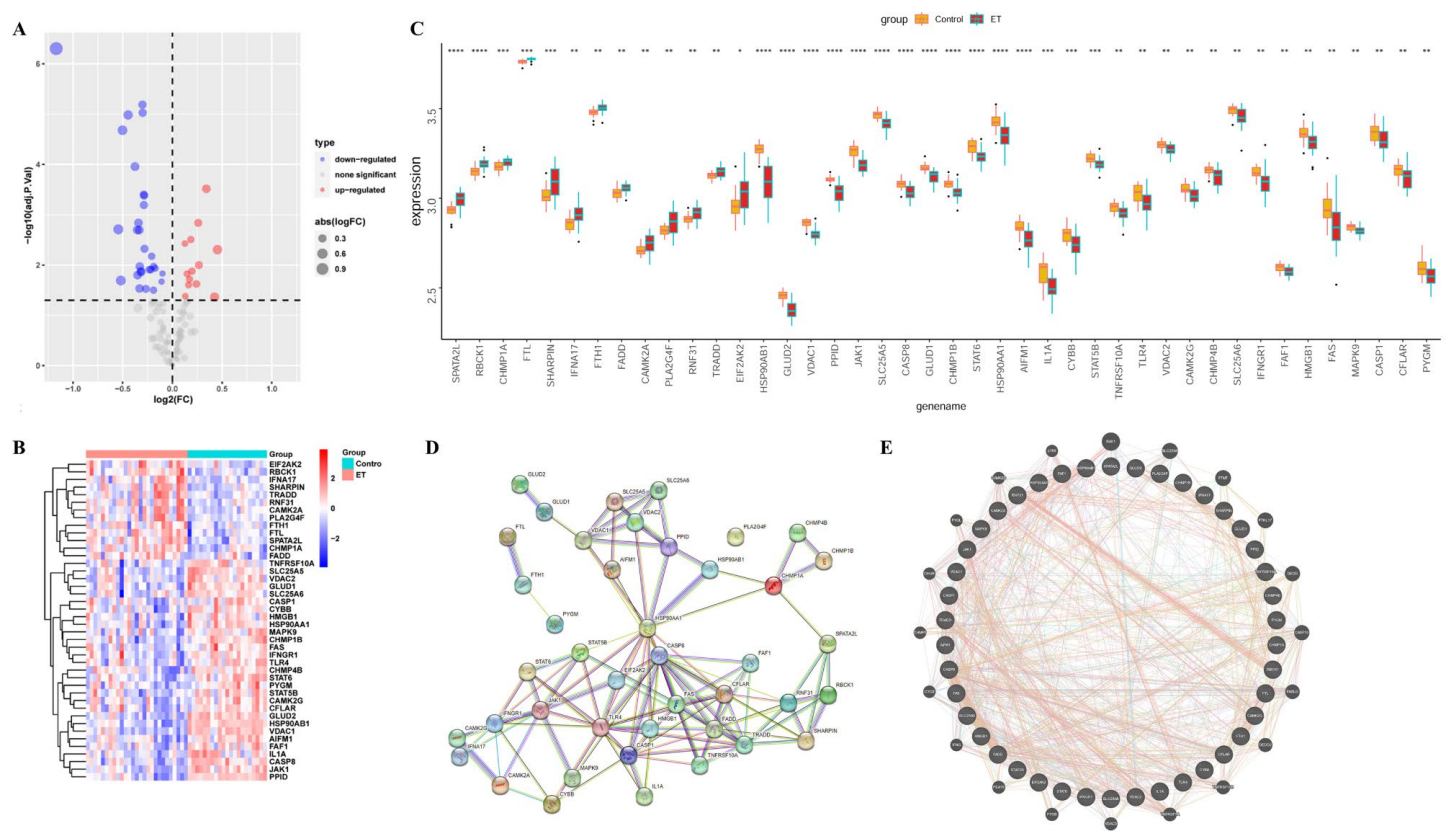
Protein interaction network construction. The distribution of 42 differential necrotic genes (**A**, **B**, **C**) was displayed in different forms. Based on STRING database (**D**) and genemania database (**E**), a differential necrotic gene protein interaction network was constructed. *P < 0.05, ** *P* < 0.01, *** *P* < 0.001

We further display volcano maps (Figs. 4A), heat maps (Figs. 4B) and between-group difference maps of 42 differential necroptosis genes for easy visualization. The STRING database and genemania database were used to study the interaction network of differential necrotic gene proteins, respectively. Multiple genes were found to be at the hub of the network, including HSP90AA1, EIF2AK2, CASP8, CASP8, CFLAR, FAS, HMGB1, TLR4, etc. (Figs. 4C). Networks show that multiple genes are closely linked in terms of physical interactions and shared protein domains (Fig. 4D-E).

### Identification of biomarkers in ET

**Fig. 5:**
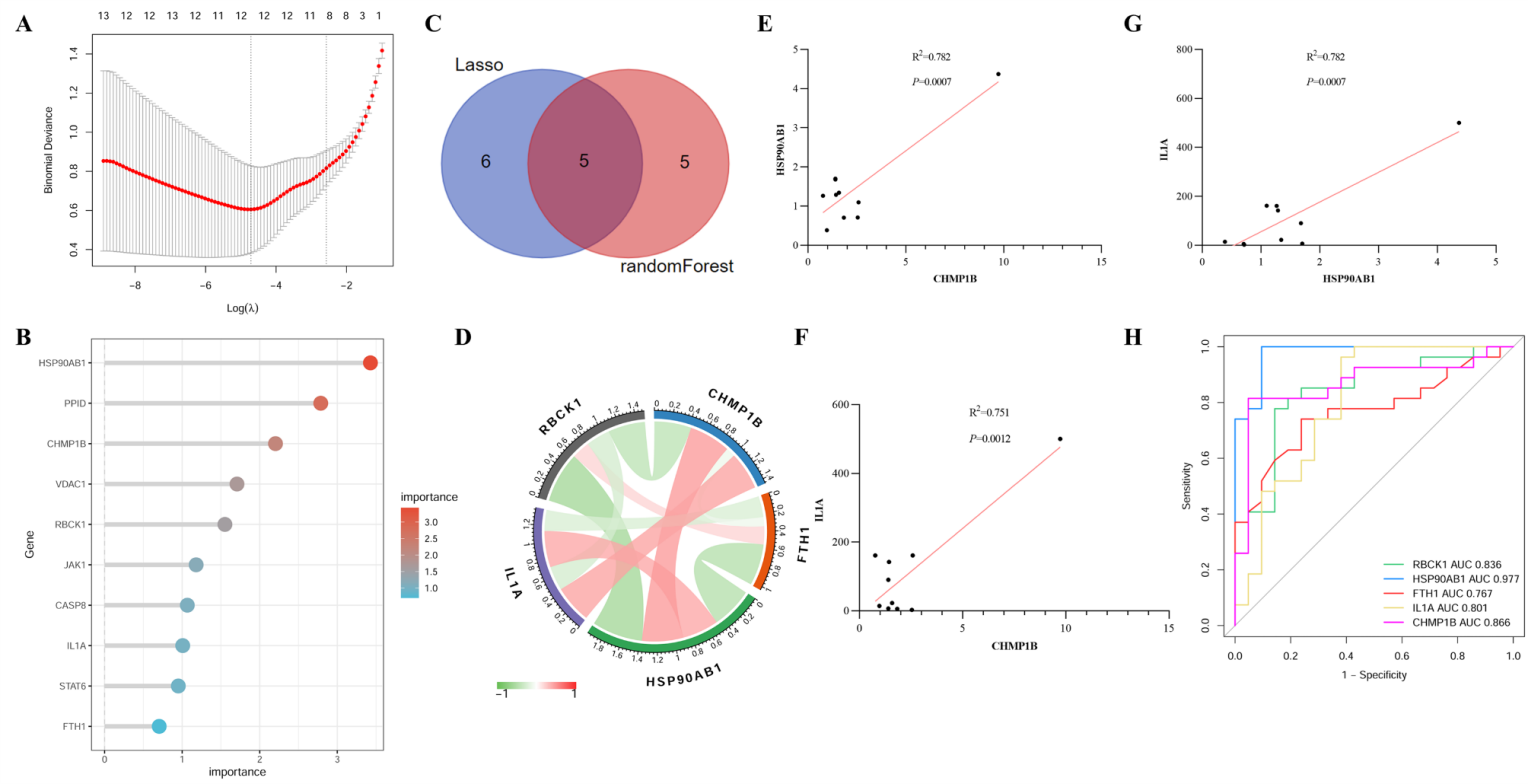
Obtain key genes for ET through data machine learning. LASSO regression analysis screened 11 genes (**A**). Random forest results selected top 10 genes (**B**). The results of machine learning overlap to obtain the most important 5 genes (**C**). Construct gene correlations, red indicates positive correlation, green indicates negative correlation, and the redder the color, the stronger the correlation (**D**). Clinical samples qRT-PCR verified that CHMP1B was strongly correlated with HSP90AB1 (**E**), CHMP1B and IL1A (**F**), and HSP90AB1 and IL1A (**G**). The ROC curve was constructed in the public dataset to assess the diagnostic accuracy of 5 pairs of ETs (**H**). ET: Essential Thrombocythemia; ROC: receiver operating characteristic; AUC: area under the ROC curve

To further narrow down the differential necrosis gene range, we used data for machine learning to further narrow the gene range, including LASSO regression and random forest machine learning. By using LASSO first to pre-select a set of potentially important features, Random Forest can then be applied to further rank and validate these features, taking advantage of its ability to handle complex relationships in the data. Eleven genes were screened by lasso regression (Fig. 5A) and 10 genes were screened by random forest machine learning (Fig. 5B). After that, 5 overlapping genes were identified as a research focus (Fig. 5C) and their interrelationships were analyzed (Fig. 5D). Combined with qRT-PCR results, the results showed that CHMP1B was strongly correlated with HSP90AB1, CHMP1B and IL1A, and HSP90AB1 and IL1A (Fig. 5E-G). Finally, ROC curves were constructed in the dataset to predict the occurrence of ET, and the results show that the minimum AUC is 0.767 and the maximum is 0.977, indicating that five differential necrosis genes may be used as biomarkers for effective diagnosis of ET (Fig. 5H).

### Biomarkers and immune cell infiltration

**Fig. 6:**
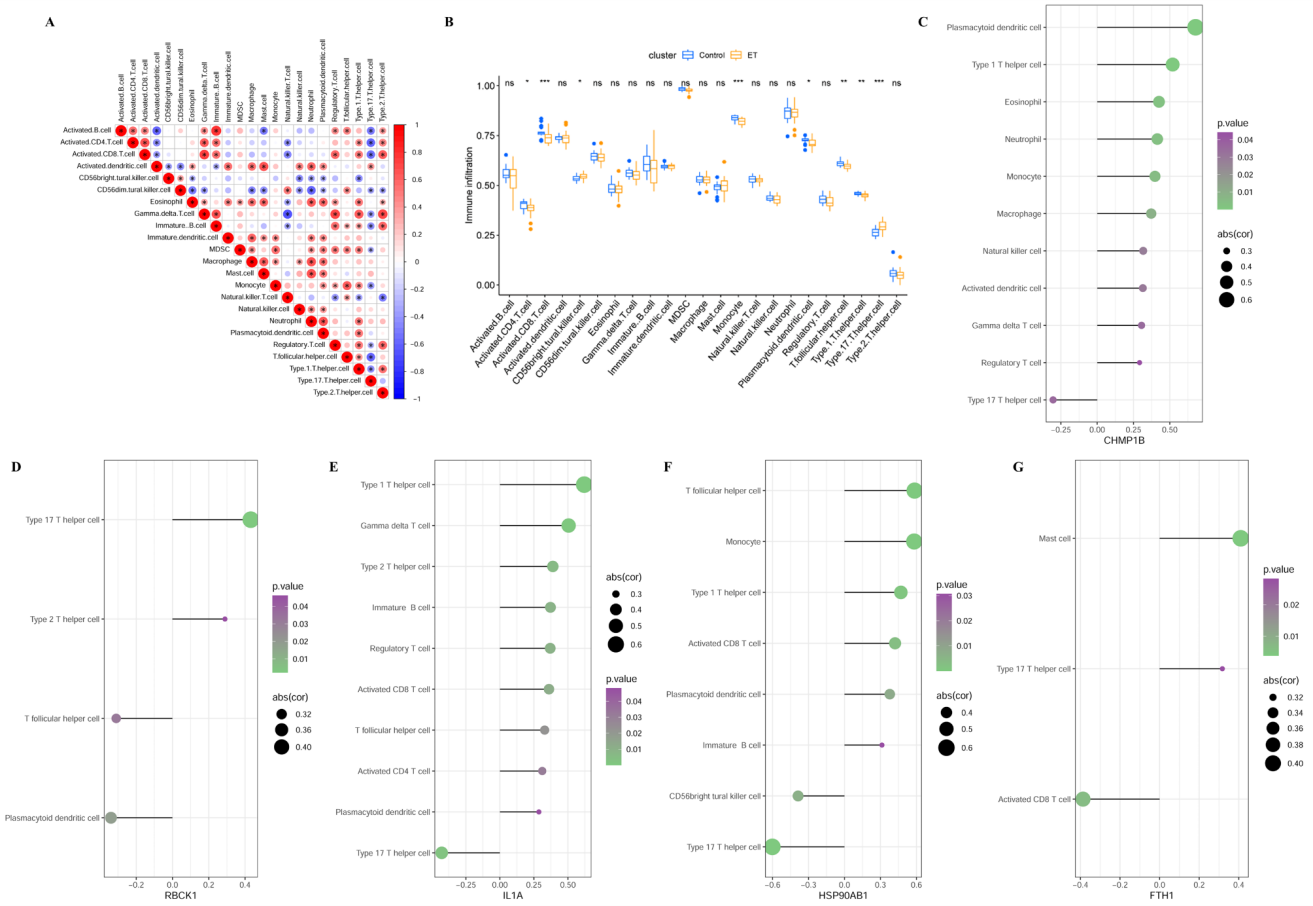
Analysis of distinctions and associations between diagnostic markers and immune infiltrating cells. Examine the correlation among immune infiltrating cells in ET. The magnitude of the colored circle represents the intensity of the connection. Red denotes a positive correlation, blue signifies a negative correlation. The more intense the connection, the redder the color shade (**A**). An * in the circle indicates *P* < 0.05. Comparison of 23 immune cell infiltration differences between ET and control subjects (**B**). Large correlation between immune infiltrating cells and 5 core genes, including CHMP1B (**C**), RBCK1 (**D**), IL1A (**E**), HSP90AB1 (**F**), FTH1 (**G**). The correlation strength is positively correlated with the size of the point, and the color of the point represents the *P* value, the greener the hue, the higher the *P* value (C-G). *P < 0.05, ** *P* < 0.01, *** *P* < 0.001. ET: Essential Thrombocythemia

First, the correlation between immune cell infiltration in ET. For example, among the 23 immune cells was explored (Fig. 6A), activated B cells were significantly positively correlated with activated CD4 T cells, activated CD8 T cells, gamma delta T cells, immature B cells, regulatory T cells, T follicular helper cells, type 2 T helper cells, and on the contrary were positively correlated with activated dendritic cells, mast cell, type 17 T helper cell is negatively correlated. Differences in immune cell infiltration were calculated using cybersport for 27 ETs and 21 control subjects (Fig. 6B), activated CD4 T cells, activated CD8 T cells, monocyte, plasmacytoid dendritic cells, T follicular helper cells, and Type 1 T helper cells in control. The expression of subjects was significantly higher than that of ET, while the expression of CD56^bright^ tural killer cell, type 17 T helper cell was the opposite. Based on the results of biomarkers and immune infiltration assays, we further analyzed the correlation of diagnostic markers with immune infiltrating cells. The results showed CHMP1B with plasmacytoid dendritic cell, type 1 T helper cell, RBCK1 and type 17 T helper cell, IL1A and type 1 T helper cell, gamma delta T cell, HSP90AB1 with T follicular helper cell, monocyte, type 1 T helper cell, FTH1, is significantly positively correlated with the Mast cell (Fig. 6C-G). These studies demonstrated that necroptosis and immune cell infiltration may be involved in the development of ET, and the positive correlation between the two suggested that necrosis may accelerate the progression of ET through driving immune infiltration and immune response.

### Single gene correlation analysis and functional enrichment analysis

**Fig. 7:**
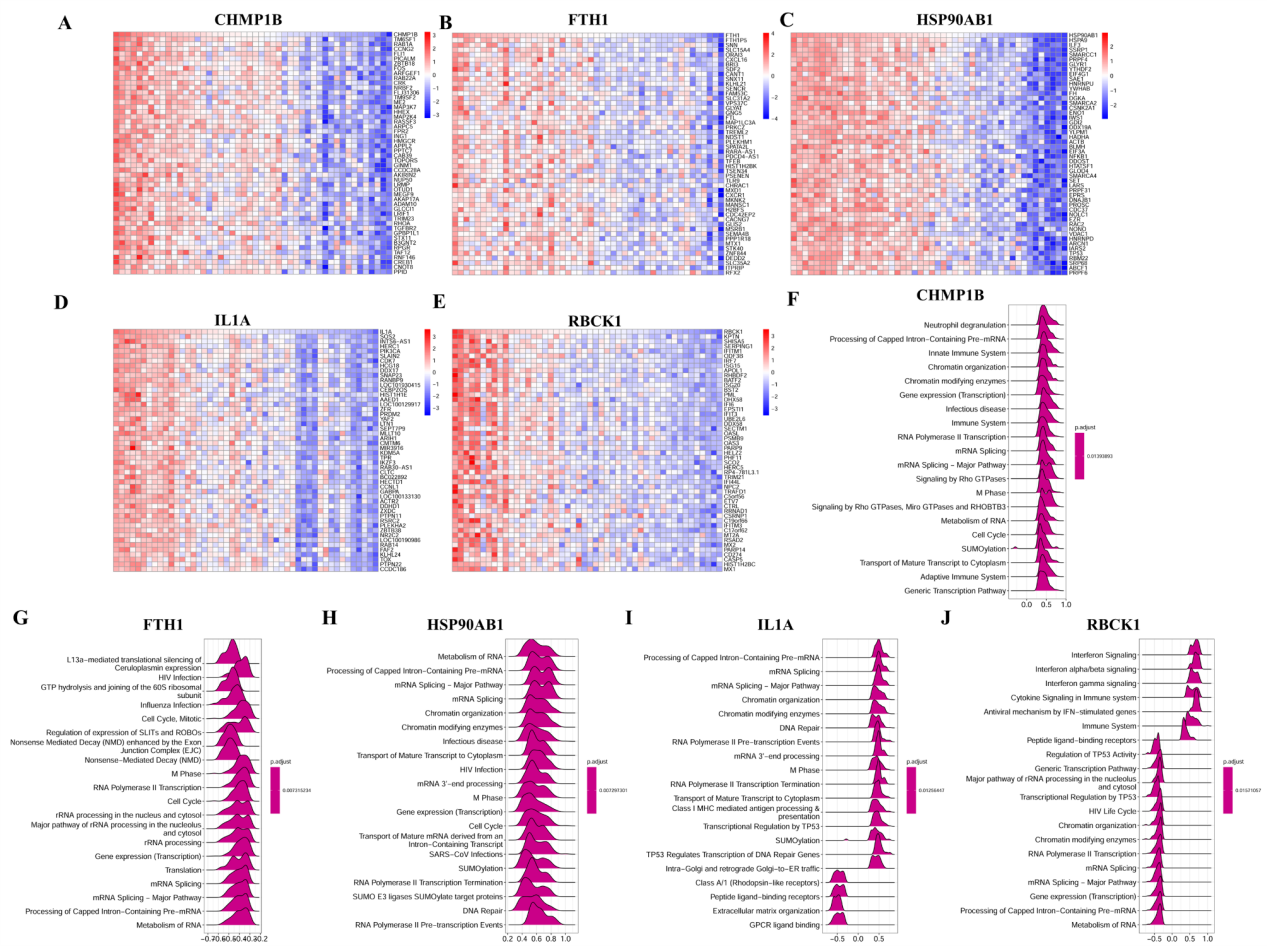
Core gene enrichment analysis. Correlation construction of 5 genes with all genes(**A**-**E**). Based on the results of single gene enrichment analysis based on the Reactome database, the abscissa represents the enrichment score, and greater than 0 indicates that the gene is positively correlated with the pathway, and vice versa (**F**-**J**). ET: Essential Thrombocythemia

To examine the potential biological function of biomarkers in ET, we performed a single-gene functional enrichment analysis according to the Reactome database. Firstly, the association between 5 necrotic genes and all genes in 48 samples in the dataset was analyzed, and the top 50 results were selected for visual display (Fig. 7A-E). Then, according to the results of gene correlation analysis, a single gene enrichment analysis was carried out and the top 20 biological functional pathways were demonstrated (Fig. 7F-J). The five genes are mainly involved in mRNA processing, pathogen infection, the immune system and other pathways.

### Exploration of upstream regulatory mechanisms of biomarkers

**Fig. 8:**
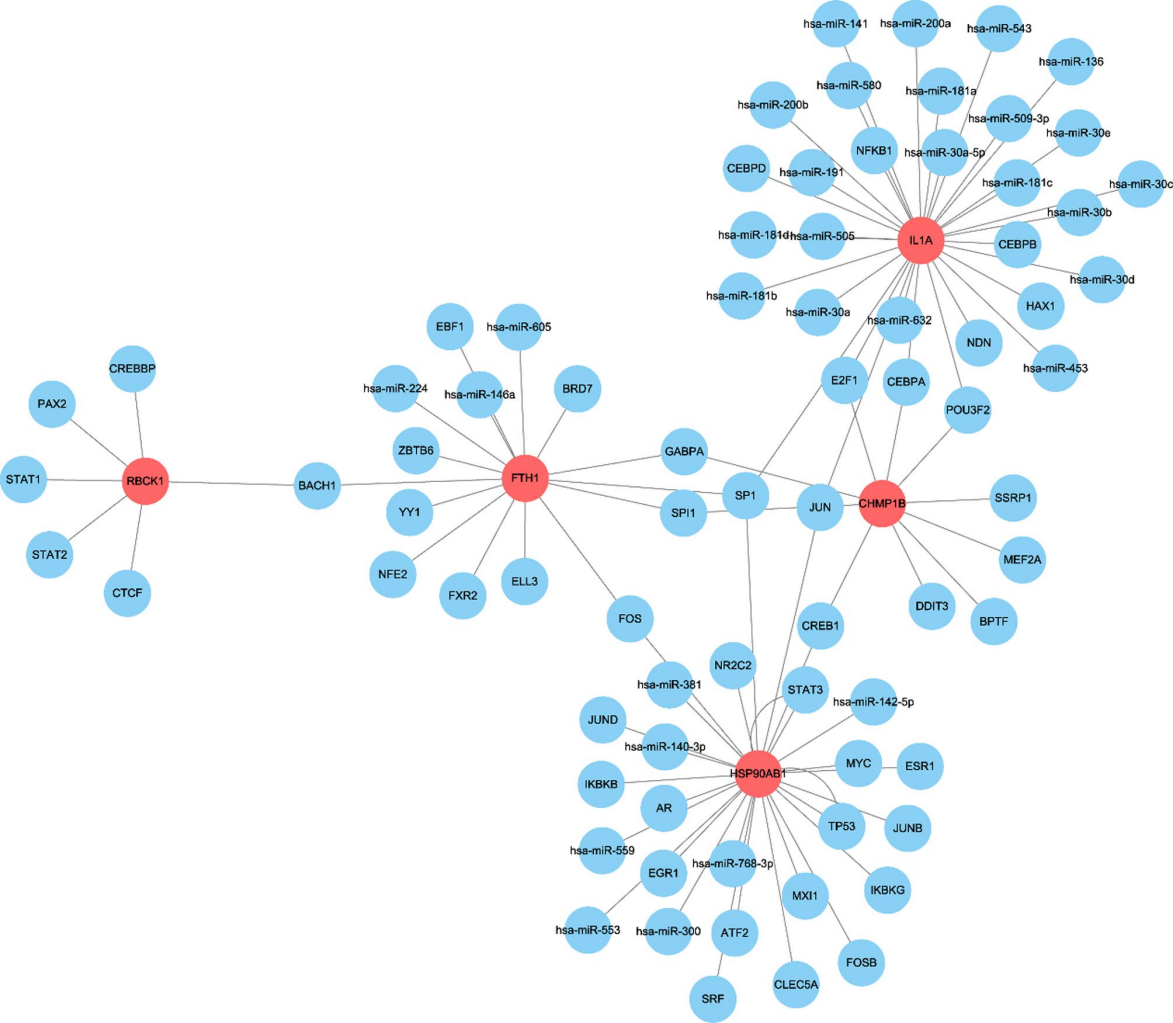
Exploration of molecular mechanisms of biomarkers. TF and miRNA regulatory network construction of five genes, red for diagnostic marker genes, blue for TF and miRNA

Regnetwork was used to explore the regulatory mechanisms of miRNA and TF upstream of genes, visualized using Cytoscape software. The results indicated that 5 genes were related to multiple TFs and miRNAs, and that one TF could regulate multiple genes (Fig. 8).

### Validation of biomarkers

**Fig. 9:**
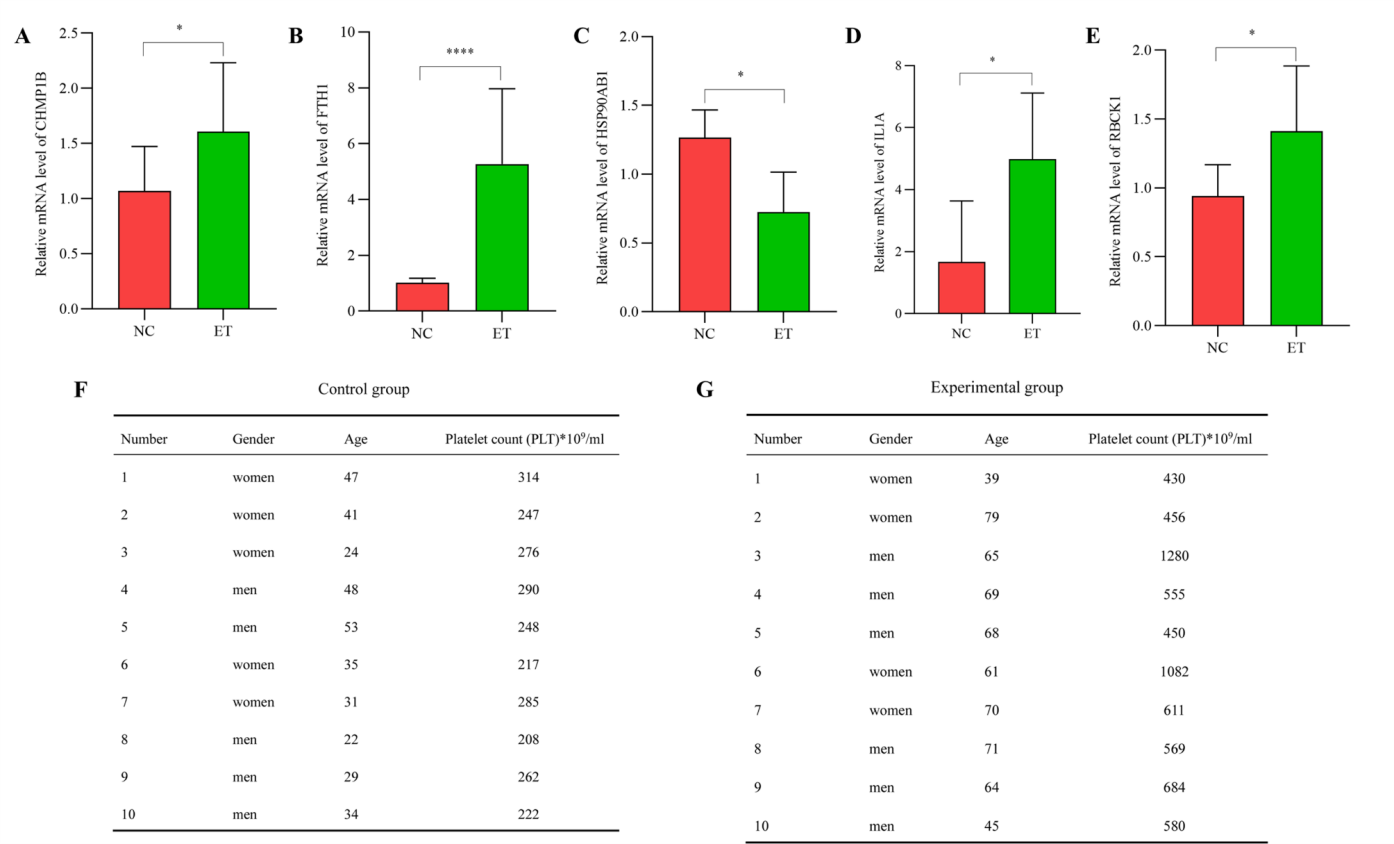
Verification of biomarker expression by qRT-PCR. The expression of CHMP1B (**A**), FTH1 (**B**), HSP90AB1 (**C**), ILA1 (**D**), and RBCK1 (**E**) in ET patients. The clinical characteristics of control group (**F**) and experimental group (**G**). *P < 0.05, ** *P* < 0.01, *** *P* < 0.001

Expression of 5 biomarkers in clinical samples was detected using qRT-PCR. With the exception of HSP90AB1 (Fig. 9C), the remaining four markers showed significant upregulation (Fig. 9A, B, D, E), indicating the reproducibility and reliability of the results. Figure 9F-G summarizes sample clinical information.

## Discussion

ET is a disease frequently encountered in clinical practice, it often occurs in older people [17]. Palpable splenomegaly, leukocytosis, abnormal karyotype and thrombosis are common concurrent symptoms [18]. Additional associated symptoms include headache, dizziness, visual disturbances, dysaesthesia and, more rarely, erythematous limb pain [1]. Low-dose aspirin is the cornerstone of therapy used to reduce the risk of thrombosis, but hydroxyurea or interferon is the most commonly used cytoreductive option for ET patients at high risk of vascular complications when treating patients at moderate and high risk [19]. Neverthless, some patients still experience rapid symptoms progression, and there is even a 4% probability of leukemia transformation [1]. Consequently, there is an urgent need for more effective and accurate diagnostic markers.

In this study, we confirmed the presence of necroptosis in ET. To our knowledge, this is the first research manuscript to report the presence of necroptosis in the pathogenesis of ET in humans. First, we integrated two GEO datasets to identify 42 differential Necroptosis genes through genetic difference analysis and Necroptosis gene sets. Enrichment analysis of these 42 genes revealed their activation in Necroptosis, influenza A, NOD-like receptor signaling pathways, neurodegenerative pathways-multiple diseases and lipids and atherosclerosis. Further by applying data machine learning to the data for biomarkers identification, we obtained 5 marker genes, including CHMP1B, FTH1, HSP90AB1, IL1A and RCBK1. GSEA based on KEGG showed that 5 genes were mainly enriched in Necroptosis, NOD-like receptor signaling pathway, and influenza A and measles infection pathways. Reactome single gene enrichment analysis mainly involved in mRNA processing, pathogen infection, immune system and other pathways. Currently, Necroptosis is regarded as an alternate cell death defense mechanism that is triggered when apoptosis is blocked, such as during pathogen infection [20, 21]. This finding is consistent with the necrotic pathway involved in the five genes and the pathogen infection pathway, and is related to the change of ET immune status.

Based on the above findings, we further examined the difference in immune cell infiltration enrichment between ET and normal samples, and found that CD56^bright^ tural killer cell and type 17 T helper cells were significantly higher expressed in ET. Studies have found that CD56^bright^ tural killer cell exhibits abnormal receptor expression and cytokine production, which is seriously associated with aplastic anemia [22]. Type 17 T helper cell is a subtype of CD4 T cell differentiation, and its differentiation is related to the differentiation of iTreg, and TGF-β is required for both subtypes of differentiation [23]. Type 17 has been shown to participate in the pathogenesis of multiple autoimmune diseases and shows a strong dependence on cellular environmental triggers [24].

This study represents the first instance of identifying an imbalanced expression of Type 17 T helper cells, and it is significantly positively correlated with the marker gene RBCK1. Regarding another subtype of CD4 T cells, Type 1 T helper cells, were remarkably reduced in ET and positively correlated with CHMP1B, IL1A and HSP90AB1 expression. Additionally, Type 1 helper cells is known to produce interferon-γ, interleukin-2, and tumor necrosis factor β and activates macrophages responsible for cell-mediated immune and phagocyte-dependent protective responses [25]. This suggests that necrotizing apoptotic cells caused by CHMP1B, IL1A, and HSP90AB1 may be recognized by type 1 help cells, thereby activating cellular immune processes in the body. Taken together, these studies imply that necroptosis may accelerate the progression of ET by driving immune infiltration and immune response.

In addition, the expression of CHMP1B, FTH1, HSP90AB1, IL1A and RCBK1 were validated in 20 clinical samples, which was consistent with datasets-derived findings. The validation groups included 10 cases of NC group (5 males and 5 females) and 10 cases in ET group (6 males/4 females, 39–79 years). The GEO dataset (GSE57793) had population characteristics, which include 9 males and 10 females in ET group (35–87 years), 21 males and 20 females in PV group (35–85 years), 3 males and 6 females in PMF groups (53–74 years). The present validation group has representativeness of samples with GEO datasets. Moreover, except for HSP90AB1, the remaining four markers showed significant upregulation, indicating the reproducibility and reliability of the results.

However, this study is subject to certain limitations. First, although diagnostic markers have been rigorously analyzed via rigorous bioinformatics analysis and qRT-PCR validation, the absence of protein-level experiments leaves the results unsubstantiated. Second, the small sample size within the datasets necessitates confirmation through larger and more prospective studies. Third, while our analysis has established a correlation between necroptosis and immune cell infiltration in ET, the specific mechanistic relationship between the two remains unproven, thus warranting further investigation.

## Conclusions

Via genetic difference analysis, with the utilization of necroptosis gene sets in conjunction with multiple machine learning algorithms, CHMP1B, FTH1, HSP90AB1, IL1A, and RCBK1 were identified as biomarkers of ET. In addition, the examination of immune infiltration demonstrated the existence of type 1 and type 17 expression disorders in ET, which exhibited significant correlations with multiple necroptosis genes. This indicates that necroptosis and immune infiltration have crucial roles in ET. Consequently, our results may potentially serve as a novel reference for the diagnosis and treatment of ET in the forthcoming years.

## Electronic supplementary material

Below is the link to the electronic supplementary material.

Supplementary Material 1

## Data Availability

All data generated or analyzed during this study are included in this article. Further enquiries can be directed to the corresponding author.

## Abbreviations

BP: Biological Process
CC: Cellular Component
ET: Essential Thrombocytosis
GEO: Gene Expression Omnibus
GO: Gene Ontology
KEGG: Kyoto Encyclopedia of Genes and Genomes
MF: Molecular Function
qRT-PCR: Quantitative Reverse Transcription-PCR
SD: Standard deviation
