# Supplementary material for: Role of necroptosis and immune infiltration in essential thrombocytosis
Source: Hereditas. 2025 Apr 14;162:62. doi: 10.1186/s41065-025-00428-1 (PMC11995491; doi:10.1186/s41065-025-00428-1)
Supplement: Supplementary file 1 — Supplementary Material 1 [file 41065_2025_428_MOESM1_ESM.docx]

**Supplementary Table 1** Oligonucleotide primer sequences used in the qRT-PCR

| **Gene** | **Forward** | **Reverse** |
| --- | --- | --- |
| GAPDH | GCACCGTCAAGGCTGAGAAC | TGGTGAAGACGCCAGTGGA |
| RBCK1 | TGCTCAGATGCACACCGTC | CAAGACTGGTGGGAAGCCATA |
| FTH1 | CCCCCATTTGTGTGACTTCAT | GCCCGAGGCTTAGCTTTCATT |
| HSP90AB1 | AGAAATTGCCCAACTCATGTCC | ATCAACTCCCGAAGGAAAATCTC |
| CHMP1B | AAAGAACTGAGTAGGAGTGCCA | TGTATCCTCGCAACTTCCATGT |
| IL1A | TGGTAGTAGCAACCAACGGGA | ACTTTGATTGAGGGCGTCATTC |
